# Supplementary figures and images for: Identification of exceptionally potent adenosine deaminases RNA editors from high body temperature organisms
Source: PLoS Genet. 2023 Mar 6;19(3):e1010661. doi: 10.1371/journal.pgen.1010661 (PMC10019624; doi:10.1371/journal.pgen.1010661)

**A.**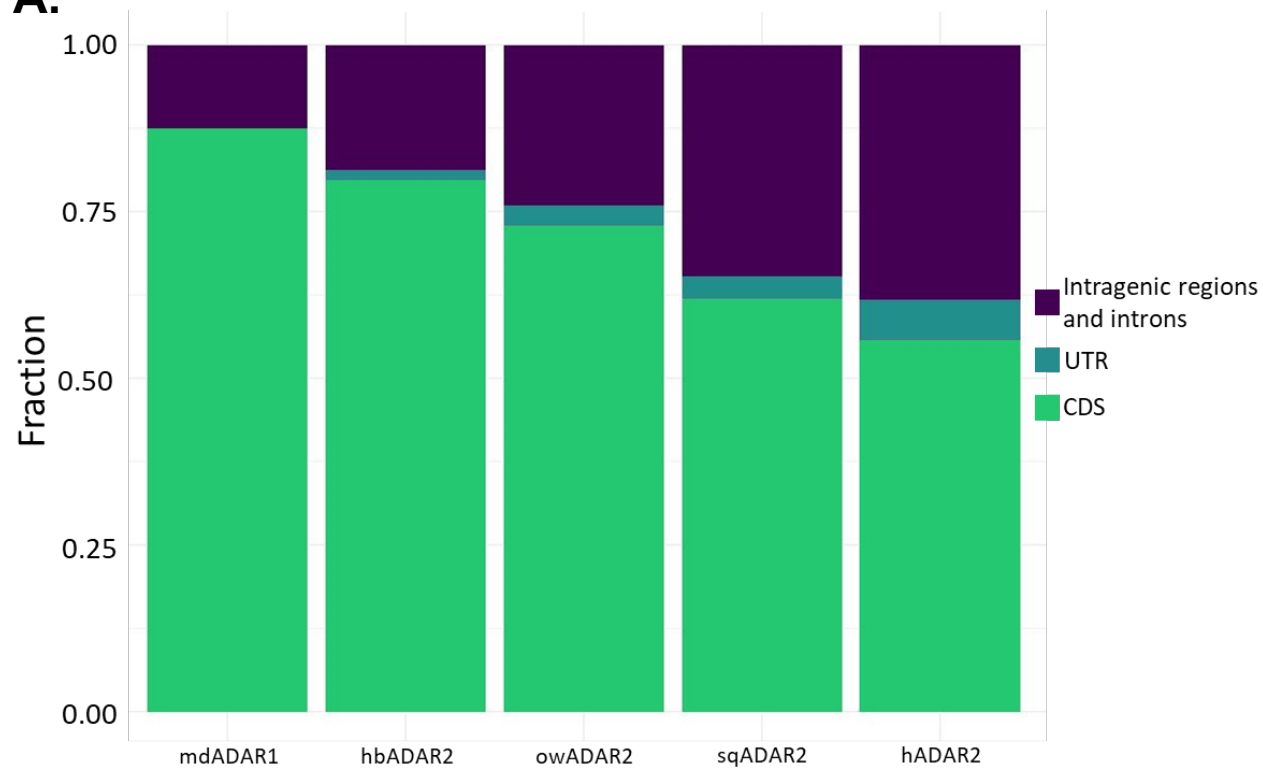**B.**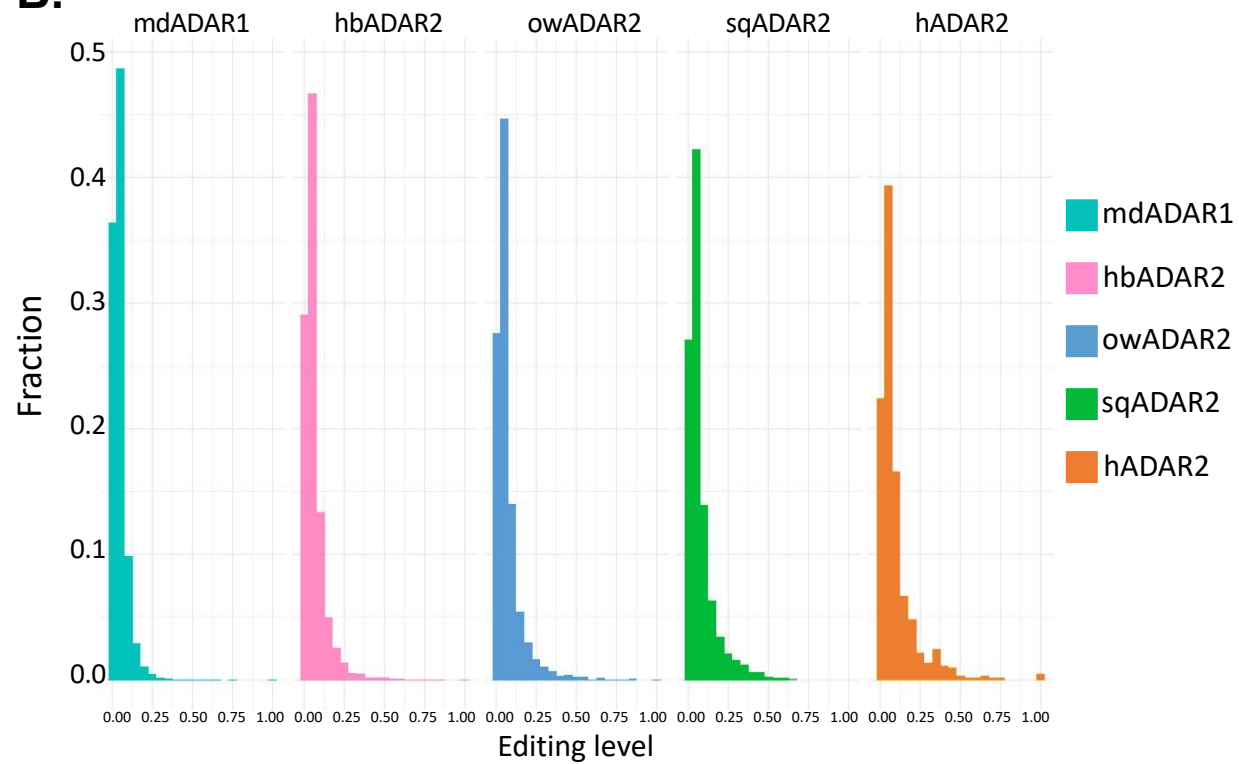

Supplement: S1 Fig — (PDF) [file pgen.1010661.s001.pdf]

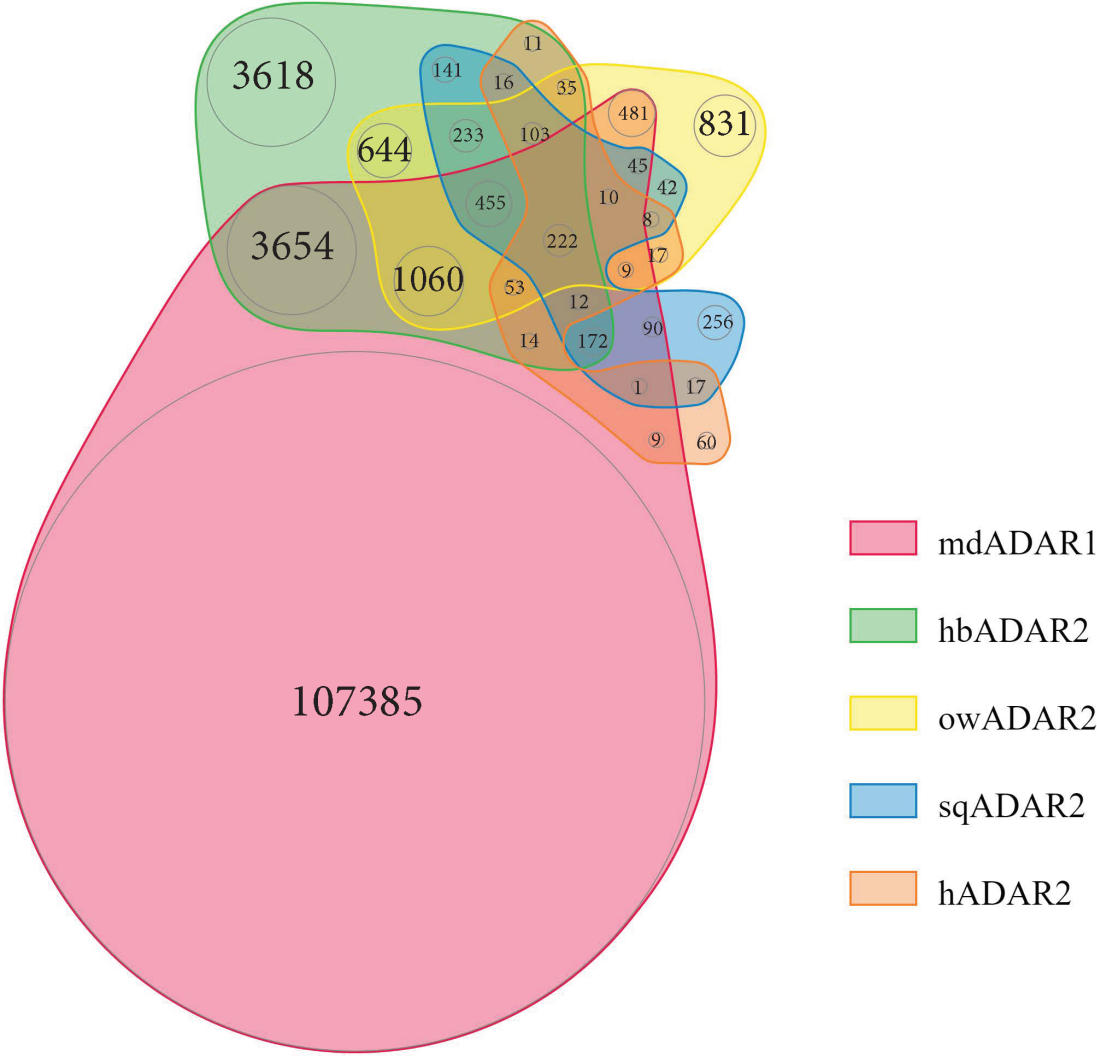

Supplement: S2 Fig — (PDF) [file pgen.1010661.s002.pdf]

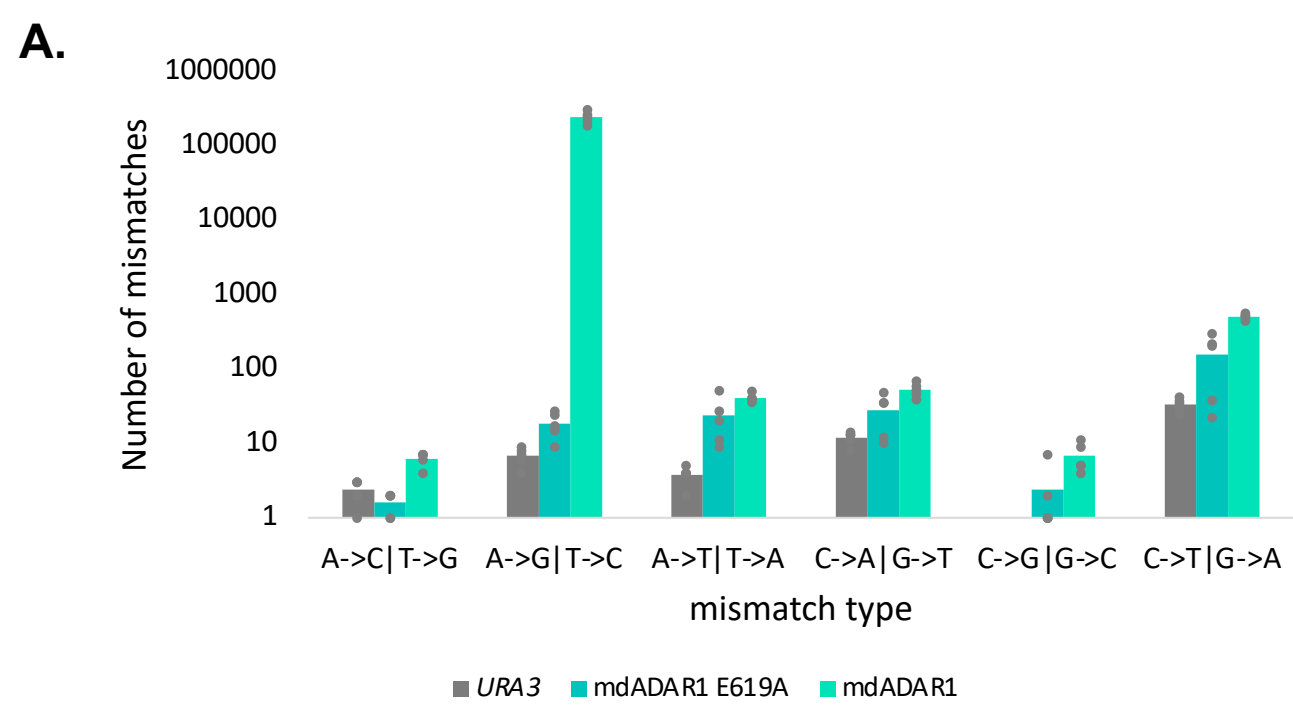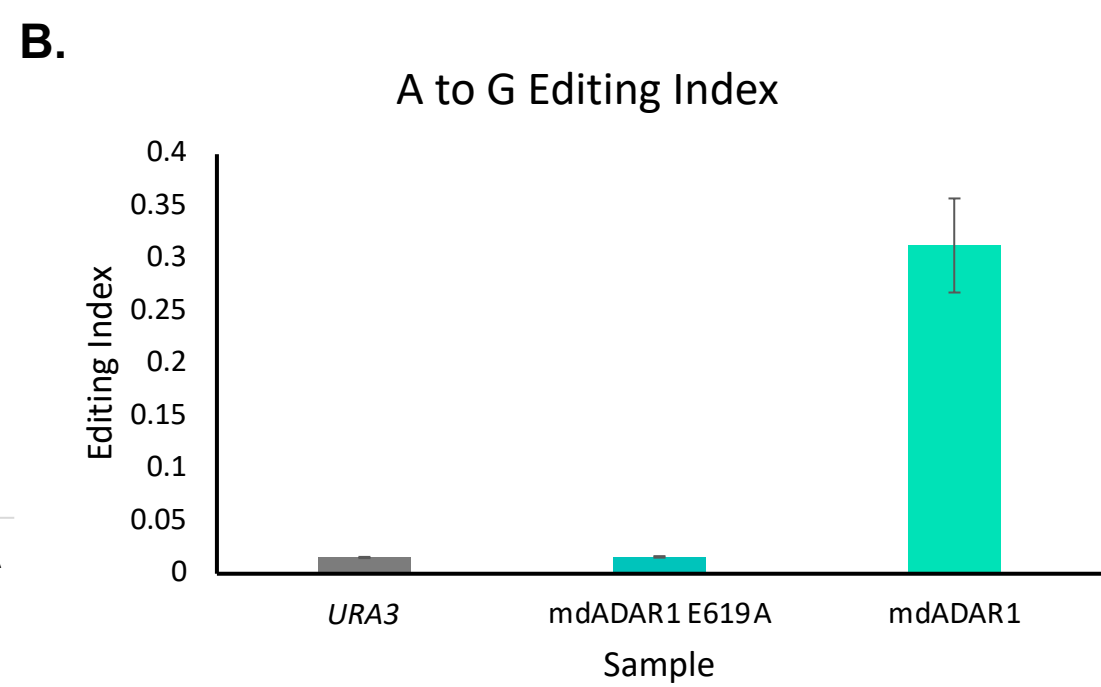

Supplement: S3 Fig — (PDF) [file pgen.1010661.s003.pdf]

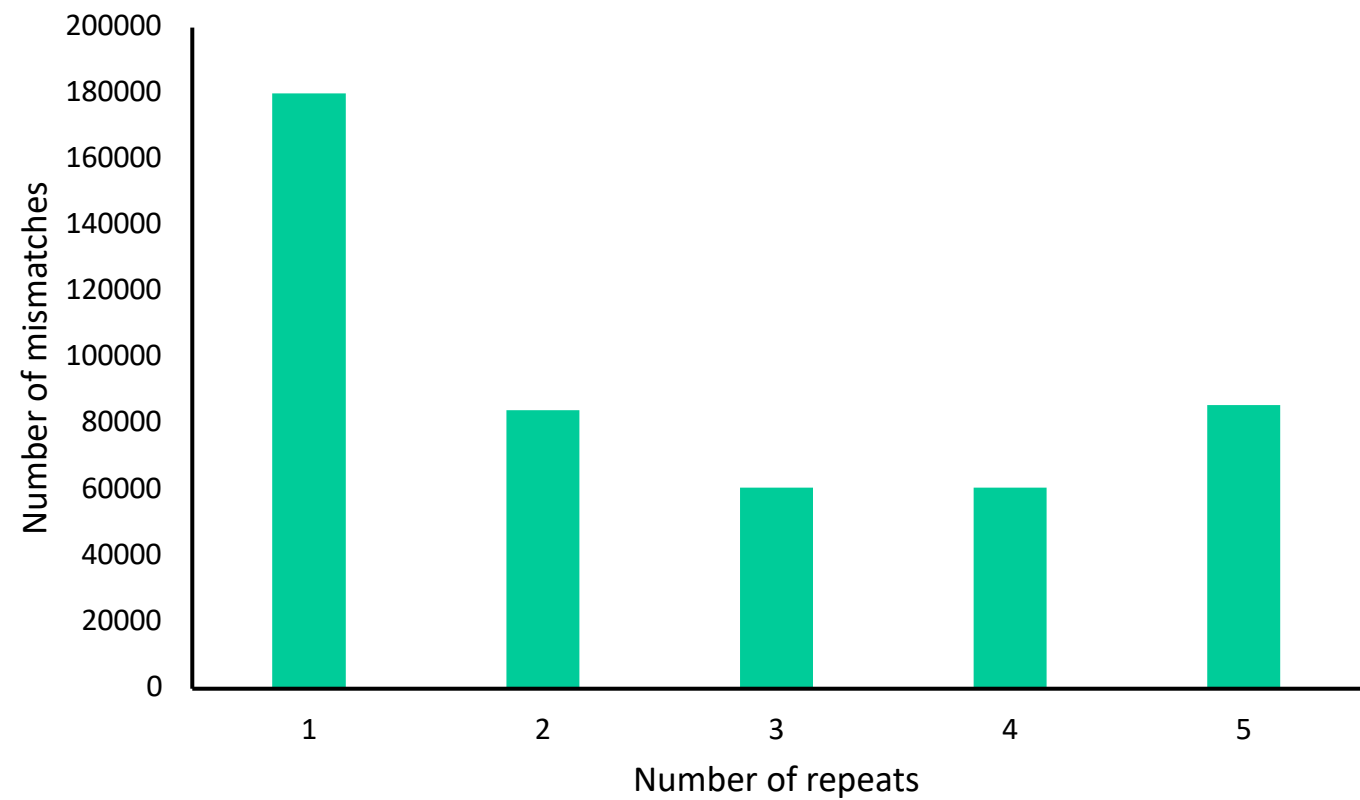

Supplement: S4 Fig — (PDF) [file pgen.1010661.s004.pdf]
