# Supplementary material for: Identification of exceptionally potent adenosine deaminases RNA editors from high body temperature organisms
Source: PLoS Genet. 2023 Mar 6;19(3):e1010661. doi: 10.1371/journal.pgen.1010661 (PMC10019624; doi:10.1371/journal.pgen.1010661)
Supplement: S5 Fig — (PDF) [file pgen.1010661.s005.pdf]

S5 Fig

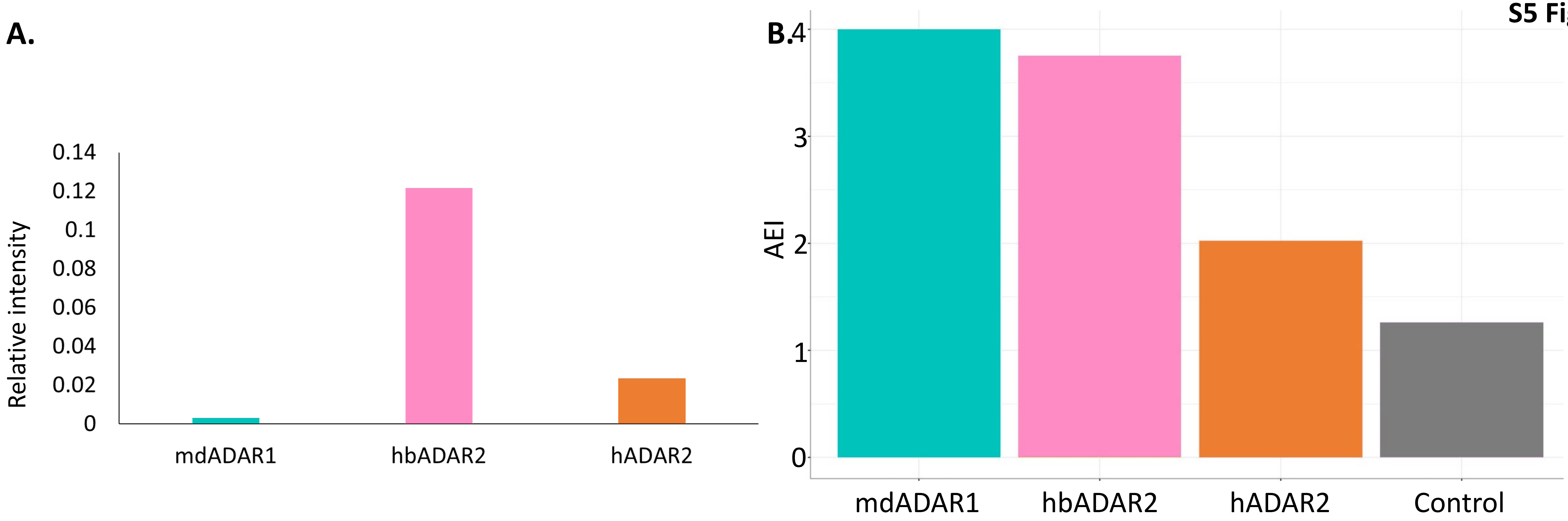


**S5 Fig. Induced expression of mdADAR1 in human HeLa cells results in increased editing levels compared to the levels seen for overexpressed hADAR2A**. **(A)** HeLa cells were cultured to ~70% confluency and transfected with either pTwist-CMVp-mdADAR1, pTwist-CMVp-hbADAR2 or pTwist-CMVp-hADAR2 plasmids. Cells were allowed to grow for 3 days in DMEM medium supplemented with FBS, L-Glutamine, Pen-Sprep-Nis, and puromycin was added for plasmid selection. Proteins were extracted from the cells, separated by SDS-PAGE and immunoblotted with anti-Flag antibody. Ponceau staining was used as loading control. Relative intensity was measured as pixel density for each sample. **(B)** RNA was extracted from the cells described in (A) and control HeLa cells. Following RNA-sequencing, the global ADAR activity was quantified by the Alu Editing Index (AEI) [1]. Although mdADAR1 protein levels were lower than hADAR2, the global editing effect was larger.

## References


1. Roth SH, Levanon EY, Eisenberg E. Genome-wide quantification of ADAR adenosine-to-inosine RNA editing activity. Nat Methods. 2019;16: 1131–1138. doi:10.1038/S41592-019-0610-9
