# Supplementary material for: Identification of exceptionally potent adenosine deaminases RNA editors from high body temperature organisms
Source: PLoS Genet. 2023 Mar 6;19(3):e1010661. doi: 10.1371/journal.pgen.1010661 (PMC10019624; doi:10.1371/journal.pgen.1010661)
Supplement: S1 Table — (PDF) [file pgen.1010661.s006.pdf]

Table S1. Yeast strains used in this study

Strain	Parental strain	Genotype	Plasmid	Origin
BY4742		*MATα; ura3Δ0; leu2Δ0; his3Δ1; lys2Δ0*	-	[1]
YSB2062	BY4742	*MATα; ura3Δ0; leu2Δ0; his3Δ1; lys2Δ0* +pYES-DEST52-mdADAR1::*URA3*	BSB474	This study
YSB3022	BY4742	*MATα; ura3Δ0; leu2Δ0; his3Δ1; lys2Δ0* +pYES-DEST52-mdADAR2::*URA3*	BSB688	This study
YSB3023	BY4742	*MATα; ura3Δ0; leu2Δ0; his3Δ1; lys2Δ0* +pYES-DEST52-hbADAR1::*URA3*	BSB689	This study
YSB2063	BY4742	*MATα; ura3Δ0; leu2Δ0; his3Δ1; lys2Δ0* +pYES-DEST52-hbADAR2::*URA3*	BSB475	This study
YSB3025	BY4742	*MATα; ura3Δ0; leu2Δ0; his3Δ1; lys2Δ0* +pYES-DEST52-owADAR1::*URA3*	BSB690	This study
YSB2064	BY4742	*MATα; ura3Δ0; leu2Δ0; his3Δ1; lys2Δ0* +pYES-DEST52-owADAR2::*URA3*	BSB476	This study
YSB2065	BY4742	*MATα; ura3Δ0; leu2Δ0; his3Δ1; lys2Δ0* +pYES-DEST52-sqADAR1::*URA3*	BSB405	This study
YSB2066	BY4742	*MATα; ura3Δ0; leu2Δ0; his3Δ1; lys2Δ0* +pYES-DEST52-sqADAR2::*URA3*	BSB393	This study
YSB2067	BY4742	*MATα; ura3Δ0; leu2Δ0; his3Δ1; lys2Δ0* +pYES-DEST52-hADAR1::*URA3*	BSB575	This study
YSB2068	BY4742	*MATα; ura3Δ0; leu2Δ0; his3Δ1; lys2Δ0* +pYES-DEST52-hADAR2::*URA3*	BSB394	This study
YSB2069	BY4742	*MATα; ura3Δ0; leu2Δ0; his3Δ1; lys2Δ0* +pYES-DEST52::*URA3*	BSB13	This study
YSB3424	BY4742	*MATα; ura3Δ0; leu2Δ0; his3Δ1; lys2Δ0* +pYES-DEST52-mdADAR1-DD::*URA3*	BSB873	This study
YSB3425	BY4742	*MATα; ura3Δ0; leu2Δ0; his3Δ1; lys2Δ0* +pYES-DEST52-mdADAR1-DD-RBM1-2-3::*URA3*	BSB874	This study
YSB3426	BY4742	*MATα; ura3Δ0; leu2Δ0; his3Δ1; lys2Δ0* +pYES-DEST52-mdADAR1-DD-RBM2-3::*URA3*	BSB875	This study
YSB3427	BY4742	*MATα; ura3Δ0; leu2Δ0; his3Δ1; lys2Δ0* +pYES-DEST52-mdADAR1-DD-RBM3::*URA3*	BSB876	This study
YSB3428	BY4742	*MATα; ura3Δ0; leu2Δ0; his3Δ1; lys2Δ0* +pYES-DEST52-mdADAR1-DD-hADAR1-RBM::*URA3*	BSB877	This study
YSB3429	BY4742	*MATα; ura3Δ0; leu2Δ0; his3Δ1; lys2Δ0* +pYES-DEST52-hADAR1-DD-mdADAR1-RBM::*URA3*	BSB878	This study

Refrences

1. Brachmann CB, Davies A, Cost GJ, Caputo E, Li J, Hieter P, et al. Designer deletion strains derived from Saccharomyces cerevisiae S288C: A useful set of strains and plasmids for PCR-mediated gene disruption and other applications. Yeast. 1998;14: 115–132. doi:10.1002/(SICI)1097-0061(19980130)14:2<115::AID-YEA204>3.0.CO;2-2
