# Supplementary material for: Identification of exceptionally potent adenosine deaminases RNA editors from high body temperature organisms
Source: PLoS Genet. 2023 Mar 6;19(3):e1010661. doi: 10.1371/journal.pgen.1010661 (PMC10019624; doi:10.1371/journal.pgen.1010661)
Supplement: S2 Table — (PDF) [file pgen.1010661.s007.pdf]

Table S2. Plasmids used in this study

Name	Content	Bacteria selection marker	Yeast selection marker	Origin
BSB474	pYES-DEST52-mdADAR1::*URA3*	Amp	*URA3*	This study
BSB688	pYES-DEST52-mdADAR2::*URA3*	Amp	*URA3*	This study
BSB689	pYES-DEST52-hbADAR1::*URA3*	Amp	*URA3*	This study
BSB475	pYES-DEST52-hbADAR2::*URA3*	Amp	*URA3*	This study
BSB690	pYES-DEST52-owADAR1::*URA3*	Amp	*URA3*	This study
BSB476	pYES-DEST52-owADAR1::*URA3*	Amp	*URA3*	This study
BSB405	pYES-DEST52-sqADAR1::*URA3*	Amp	*URA3*	This study
BSB393	pYES-DEST52-sqADAR2::*URA3*	Amp	*URA3*	This study
BSB575	pYES-DEST52-hADAR1::*URA3*	Amp	*URA3*	This study
BSB394	pYES-DEST52-hADAR2::*URA3*	Amp	*URA3*	This study
BSB13	pYES-DEST52::*URA3*	Amp	*URA3*	Invitrogen catalog number 12286019
BSB873	pYES-DEST52-mdADAR1-DD::*URA3*	Amp	*URA3*	This study
BSB874	pYES-DEST52-mdADAR1-DD-RBM1-2-3::*URA3*	Amp	*URA3*	This study
BSB875	mdADAR1-DD-RBM2-3::*URA3*	Amp	*URA3*	This study
BSB876	pYES-DEST52-mdADAR1-DD-RBM3::*URA3*	Amp	*URA3*	This study
BSB877	pYES-DEST52-mdADAR1-DD-hADAR1-RBM::*URA3*	Amp	*URA3*	This study
BSB878	pYES-DEST52-hADAR1-DD-mdADAR1-RBM::*URA3*	Amp	*URA3*	This study
BSB879	pTwist-CMVp-mdADAR1-Puro	Amp	Puromycin	This study
BSB880	pTwist-CMVp-hbADAR2-Puro	Amp	Puromycin	This study
BSB881	pTwist-CMVp-hADAR2-Puro	Amp	Puromycin	This study
