# Supplementary material for: Identification of exceptionally potent adenosine deaminases RNA editors from high body temperature organisms
Source: PLoS Genet. 2023 Mar 6;19(3):e1010661. doi: 10.1371/journal.pgen.1010661 (PMC10019624; doi:10.1371/journal.pgen.1010661)
Supplement: Attachment — Submitted filename: PLOS Genetics-response to the reviews letter-31.12.22.pdf [file pgen.1010661.s009.pdf]

PLOS Genetics (PGENETICS-D-22-00838)
January 2022

Dear Heather,

We thank you and the reviewers for the insightful comments and suggestions regarding our Research Article entitled “Identification of exceptionally potent adenosine deaminases RNA editors from high body temperature organisms” (PGENETICS-D-22-00838). In response to these comments, we have added new data, improved the text, and corrected mistakes. We believe that we have satisfactorily addressed all the concerns raised by the referees, and hope that you will find the revised version suitable for publication in PLOS Genetics.
In the following, please find our point-by-point response to each of the reviewers’ concerns.

Please do not hesitate to contact us if more information is required.

Best regards
Shay Ben Aroya and Eli Eisenberg

Response to Reviews

The reviewers’ comments appear in blue font, followed by our responses in black font. Some of the comments made by the three reviewers overlap, and thus there is some redundancy in our responses.

Reviewer #1:

1. It is unclear to me why in Figure 1D the authors focused on owADAR2 and not OWADAR1, which seems to be more imperative. Figure 1D should be better explained and why the authors chose to focus on these three proteins.

As mentioned in the text, although owADAR1 seemed to have an effect on yeast viability, we couldn’t validate its protein expression by WB analysis. For this reason, we decided to focus on other ADARs that exhibited an effect on viability and were detected.

2. In which regions of the yeast genome editing occur? As yeast does not contain many introns and ORFs tend not to have dsRNA structures. It will be interesting to compare editing regions in the original organism to the new sites in yeast.

As the Reviewer predicts, most sites reside within ORFs, as expected for yeast. This situation is different than editing in the original species, where most sites reside in non-coding repetitive elements. The distribution of sites by regions is presented in the new Supp. Figure. 1.

3. The hypothesis is that dsRNA structures are less stable at high temperatures and therefore are less edited. Specifically, in hADAR2 and hbADAR2. I would like to see specific examples. Concentrating on editing sites in more stable dsRNA structure, these sites should still be edited at high temperatures while less stable dsRNA should lose their editing. Showing the RNA folding experimentally would be great but this can be done bioinformatically by estimating the dG.

We thank the reviewer for this comment. As suggested, we took a comprehensive bioinformatics approach, and used RNAStructure to predict the secondary structure of the RNA sequence surrounding all sites edited by hADAR2 (reported in Fig. 5A). To compare the stability of structures between different sites we folded all of them at 30^0C and looked at the estimated dG for the substructure surrounding the editing site (typically an imperfect hairpin). We found that sites edited at higher T have a significantly more stable structure as measured by dG(30^0C), in support of our hypothesis.

These newly generated data are included in the manuscript (Figure 6C).

4. The last point is also important because of the difference in the preference of the editing enzymes, for example, hADR1 and hADAR2. It is possible that one enzyme (mallard) edits every site in a dsRNA (or even ssRNA) because of preference and not because of high temperatures and the others target specific structures that could change in variable temperatures.

We completely agree. Mallard duck is adapted to edit its necessary editing sites despite the higher temperature. This could occur through changes in the sequence surrounding the editing site that make the structures more stable, or through changes in the enzyme's preference (as suggested by the referee).

This is explained in the main text, as follows:
"Accordingly, maintaining editing at the desired level may require adaptation of the editing substrate to the ambient temperature through mutations that modify the RNA structure stability [55].
In parallel, homoeothermic species could also adapt the editing enzymes to their body temperature, changing their dsRNA binding and the catalytic activity to compensate for inter-species differences in body temperatures. We therefore suggest that species evolved to live with higher core body temperatures have developed ADAR enzymes that target weaker dsRNA structures and would therefore be more potent than other ADARs (compared at equal temperatures). "

5. I would like to see statistics (p-value) for Figure 5, specifically 5B. These are biological replicas and it shouldn't be a problem.

We thank the reviewer for noticing this omission. P-values were added to Figure 6A (formerly 5A).

Reviewer #2:

Major points

1. The authors propose that hummingbird and mallard-duck ADARs may target weaker dsRNA structures and therefore be more potent than other ADARs. Thus, the authors may compare the secondary structures surrounding the editing sites targeted by ADARs from the five species to confirm this claim.

We thank the reviewer for this comment. As suggested, we took a comprehensive bioinformatics approach, and used RNAStructure to predict the secondary structure of the RNA sequence surrounding all sites edited by the different ADAR enzymes (reported in Fig. 3A). We then compared the estimated dG for the

substructure surrounding the editing sites (usually an imperfect hairpin), and found that the potency of the different ADAR enzymes (as measured by the number of sites edited) correlates well with the stability of the edited structures. In particular, structures edited by mdADAR1 are significantly less stable that those edited by hADAR2, in agreement with our proposed model.

These results are shown in a new panel – Figure 3C – and we have added the following to the main text:
"We wanted to further test our hypothesis that the increased number of editing sites in species with higher core body temperatures is due to the fact that these species may have developed ADAR enzymes that target weaker dsRNA structures. We thus looked at the thermodynamic stability of the secondary structures surrounding the editing sites detected per species (Methods). As expected, enzymes editing more sites have edited sites with a lower stability (Figure 3C). In particular, structures edited by mdADAR1 are significantly less stable that those edited by hADAR2, in agreement with our proposed model."

2. For RNA base editing using exogenously expressed ADARs, we typically fused the ADAR's catalytic domain with a λN peptide, a SNAP-tag, or a Cas protein (dCas13), and a guide RNA was designed to recruit the enzyme to the specific site. To achieve high on-target editing efficiency, we need a catalytic domain with high editing efficiency. In this study, it is unclear whether the high editing efficiency of hummingbird and mallard-duck ADARs is due to the less stringent structural requirement of their dsRNA binding domains or the highly active catalytic domains. Thus, the authors need to show that the catalytic domain of hummingbird or mallard-duck ADAR has high editing efficiency. e.g. a comparison of the editing efficiency between dCas13-human ADAR catalytic domain and dCas13- mallard-duck catalytic domain is essential for this study.

We thank the reviewer for this helpful suggestion. To identify the domains that govern elevated potency of mdADAR1 we first carried out domain deletion experiments and compared the growth of yeast strains expressing mdADAR1 with its (a) first (b) first and second (c) all three dsRBDs deleted (new Figure 4). We found that the growth rate of these strains was similar to the wt control, implying that the combination of the DD and all three dsRBDs is important for editing.

Next, to determine if the unique editing capability of mdADAR1 is due to its deaminase domain (DD), its dsRBDs, or both, we have compared the growth of yeast strains expressing the following two hybrid enzymes: (i) mdADAR1 DD combined with human-dsRBDs1,2,3 (md-DD-hRBD1-3), and (ii) Human ADAR1-DD combined with the md-dsRBDs1-3 (hADAR1-DD-mdRBDs1-3). We found that the growth of the hADAR1-DD-mddsRBDs1-3 strain was comparable to the wt, and growth was partially impaired in the mdDD-hdsRBD1-3 strain, but not as much as for the full mdADAR1 strain. These results imply that the catalytic domain has a larger contribution to mdADAR1 potency.

Commented [EE1]: Correct?

Commented [SBa2R1]: Yes

These results are presented in new Figure. 4.

3. Since RNA base editing is mainly applied to mammalian systems, particularly in humans, the authors may express hummingbird and mallard-duck ADARs in human cells and examine their editing profiles/efficiencies.

We thank the reviewer for this helpful suggestion. We have expressed mdADAR1, hbADAR2 and hADAR2 in human HeLa cells, and compared their editing activity. As expected, the first two enzymes exhibit a much stronger editing activity as compared to hADAR2. These new results are presented in the new Supp. Fig. 5.

Minor points

1. The editing sites may be better characterized. e.g. the editing level distribution and the genic location of the editing sites can be shown.

The distribution of editing levels and the genic location (i.e. the fraction of sites found in ORFs) for all sites reported in Fig. 3A are provided in the new Supp. Figure 1.

2. Figure 1A is somehow misleading. It seems that the authors inserted 5 genes into one plasmid.

Taking into consideration this comment, we have improved Figure 1A to resolve this problem.

Reviewer #3:

The authors next focus on mdADAR1 and hbADAR2, the enzymes that produce the greatest editing levels in yeast, and using human ADAR2 (hADAR2) as control, they perform the same experiments in yeast grown at different temperatures (25°C, 30°C, 34°C). As I understand it, the stated goal is to test the idea that mdADAR1 and hbADAR2 have higher editing levels because they can edit dsRNA that is less stable, and thus should have higher levels of editing than hADAR2 at higher temperatures. Although the authors see that temperature has a big effect on editing levels in yeast expressing hADAR2, there is little effect of temperature on editing when hbADAR2 or mdADAR1 are expressed, although the authors note in the Figure 5 legend: "Res-scanner detection of editing sites (Methods) demonstrates a clear negative correlation between the growth temperature and editing activity for hADAR2 and hbADAR2."

The data shown in Figure 5 do not support the above statement. Evaluating the data shown in Figure 5 with a student's t-test is warranted, and may indeed support this statement, at least for hADAR2.

P-values were added to Fig. 6A (formerly 5A). The substantial decrease in the number of mismatches upon increasing the temperature from 25°C to 30°C is significant for both hADAR2 and hbADAR2.

That said, an effect of temperature on hADAR2 editing levels, without an effect on those from hbADAR2 or mdADAR1 might be expected if the authors' hypothesis that the latter enzymes can edit dsRNA that is less stable is true. However, additional data would be necessary to support this hypothesis.

In summary, I am convinced that the authors have identified at least two ADARs that appear to be highly active, and thus possibly useful for improving therapeutic uses of ADARs, but the observations provide little support for the idea that the differential effects are because these ADARs are editing less stable structures. In this regard unless additional data are provided, the authors should tone-down statements about this, including the sentence in the Introduction that reads: "We provide evidence that these birds which evolved to live with higher core body temperatures have developed ADAR enzymes that target weaker dsRNA structures, and would therefore be more potent than other ADARs (compared at equal temperatures)."

Importantly, the authors already have data that could be mined to test their hypothesis. For example, the authors have mapped a huge number of editing sites, and if their hypothesis is correct, the editing sites unique to mdADAR1 shown in Supplementary Figure 1 would be predicted to occur in less stable structures. There are several algorithms that could make a start in determining whether these sites occur in less stable structures.

We have now added new data to support our hypothesis that hbADAR2 and mdADAR1 edit less stable structures. We took a comprehensive bioinformatics approach, and used RNAStructure to predict the secondary structure of the RNA sequence surrounding all sites edited by the different ADAR enzymes (reported in Fig. 3A). We then compared the estimated dG for the substructure (usually a hairpin with a few mismatches) surrounding the editing sites and found that the potency of the different ADAR enzymes (as measured by the number of sites edited) correlates well with the stability of the edited structures. In particular, structures edited by mdADAR1 and hbADAR2 are significantly less stable that those edited by hADAR2, in agreement with our proposed model.

We have added the following to the main text:

"We wanted to further test our hypothesis that the increased number of editing sites in species with higher core body temperatures is due to the fact that these species may have developed ADAR enzymes that target weaker dsRNA structures. We thus looked at the thermodynamic stability of the secondary structures surrounding the editing sites detected per species (Methods). As expected, enzymes editing more sites have edited sites with a lower stability (Figure 3C). In particular, structures edited by mdADAR1 are significantly less stable that those edited by hADAR2, in agreement with our proposed model."

In truth, from the data shown, it is impossible to deconvolute changes in editing levels from changes that might occur because certain enzymes have evolved to have a specific optimal temperature. One way to specifically address this would be to choose one temperature and perform in vitro experiments with the various enzymes to compare their ability to edit several different dsRNAs designed to have different thermodynamic stabilities.

We agree that this additional experiment would be interesting. However, it should be noted that we did express all enzymes at the same temperature ($30°C$) in the same yeast system, which presents a large pool of structures of all strengths. Indeed, we see that at the same temperature, hADAR2 edits the more table structures while mdADAR1 edits also the much weaker ones (see Fig. 3C).
